# Cerebro Spinal Meningitis and Its Treatment

**Published:** 1888-05

**Authors:** A. S. Kirk


### CEREBRO-SPINAL MENINGITIS AND ITS TREATMENT.

It was in 1872 that I first met with cerebro-spinal meningitis, and I would say that the disease baffled all the skill that I could command. I was then practicing medicine some twelve miles east of this place (Louisville, Miss.) It was about the last of January, or first of February. The weather was exceedingly changeable, a great deal of sleet and rain, with a rise and fall of the temperature, generally every twenty-four hours.

I was called to see Mr. T.'s family, consisting of himself, wife and six or seven children. Two of his children were first taken sick, and died very quickly. The next that were to meet their fate were Mr. and Mrs. T., and then two more of the children were called to cross over the dark river. In the meantime several renters or tenants, both white and black, were visited by the destroying angel. I called in two other physicians, and we did all that we could, but death was in the land and it appeared that the blood was not on the door.

*Symptoms.*—In giving the symptoms I cannot do better than quote "Dickson's Elements of Medicine," with the addition of some symptoms that I will italicize. "It usually invades with a chill, succeeded with great febrile excitement, the headache being intense from the first, with severe pain down the back, and in the limbs. The countenance often flushed, expresses surprise, or wildness and terror; there is vertigo; the pupils of the eyes are generally dilated; sometimes, however, they are contracted, and, it is affirmed, may be in opposite states, one dilated and the other contracted. The patient is very restless, moaning or uttering plaintive cries; the breathing, at first hurried, soon becomes slow and oppressed, and even stertorous; the speech thick and inarticulate.

After an uncertain, but brief period, tetanoid spasms come on, being readily excited; there is hyperesthesia, with muscular rigidity. Opisthotonos is the usual form, but it is sometimes p eurothotonos or emprosthotonos. Tympanitis and constipation are present, with palsy of the bladder and lower extremities.

In the last stage there is coma; the tongue is dark and dry, with sordes on mouth and teeth; the bladder is distended, and constant stillicidium of urine, and involuntary fecal discharges take place."

*First Stage.—Tongue dark in centre and red around the edges, great pain in one finger or toe, engorgement of the lungs after six or eight hours.*

*Treatment.*—The following I find from my note book, the treatment adopted: Calomel, Dover's powders and quinine, with spirits of turpentine applied to the neck, and ironed in with a flat iron, as warm as the patient could bear it. In several cases I tried the actual cautery to the neck and spine without any effect.

The above was about the treatment we used. The last case was a negro man, whose symptoms were about those enumerated above. My experience was that the treatment we had used was worse than nothing, as all the preceding cases had been fatal. I concluded I would give him one dose and risk the consequences. I therefore gave him 60 grains of calomel. This was at night and I did not see my patient until the next morning, when to my surprise, I found him a great deal better. He was free from pain, could use his head, and had but little fever. The next day I gave quinine in 10 grain doses, every six hours, and discharged the case, his recovery being rapid. I have met with a few sporadic cases since, and have relieved my patients in every case.

When called to a case of cerebro-spinal meningitis of an adult, I commence treatment by giving calomel (20 grains) in a teaspoonful of balsam copaiba, with 30 drops of tincture gelsemium, and repeat the dose in two and a half hours, giving quinine in 10 grain doses, commencing at 4 o'clock in the morning, every two hours until I have given 30 grains. The fever is generally lower from 4 to 8 o'clock in the morning. If fever should rise, I give 20 drops of tincture of gelsemium every one and a half hours, also copaiba every six hours. If the lungs are engorged, I apply a fly plaster over each lung, let it draw well, and then apply a mush poultice to the blistered surface. If the patient is restless give some Dover's powders until sleep is produced.

By giving the balsam copaiba at the commencement of the disease we have no trouble with the urinary organs.—*Dr. A. S. Kirk, in Medical World.*

